# Integrated whole‐exome and bulk transcriptome sequencing delineates the dynamic evolution from preneoplasia to invasive lung adenocarcinoma featured with ground‐glass nodules

**DOI:** 10.1002/cam4.7383

**Published:** 2024-06-12

**Authors:** Dong Zhou, Yan‐qi Li, Quan‐xing Liu, Xu‐feng Deng, Liang Chen, Man‐yuan Li, Jiao Zhang, Xiao Lu, Hong Zheng, Ji‐gang Dai

**Affiliations:** ^1^ Department of Thoracic Surgery Xinqiao Hospital, Third Military Medical University (Army Medical University) Chongqing China

**Keywords:** ground‐glass nodule, lung adenocarcinoma, RNA‐Seq, tumor immune environment, whole‐exome sequencing

## Abstract

**Objective:**

The genomic and molecular ecology involved in the stepwise continuum progression of lung adenocarcinoma (LUAD) from adenocarcinoma in situ (AIS) to minimally invasive adenocarcinoma (MIA) and subsequent invasive adenocarcinoma (IAC) remains unclear and requires further elucidation. We aimed to characterize gene mutations and expression landscapes, and explore the association between differentially expressed genes (DEGs) and significantly mutated genes (SMGs) during the dynamic evolution from AIS to IAC.

**Methods:**

Thirty‐five patients with ground‐glass nodules (GGNs) lung adenocarcinomas were enrolled. Whole‐exome sequencing (WES) and transcriptome sequencing (RNA‐Seq) were conducted on all patients, encompassing both tumor samples and corresponding noncancerous tissues. Data obtained from WES and RNA‐Seq were subsequently analyzed.

**Results:**

The findings from WES delineated that the predominant mutations were observed in EGFR (49%) and ANKRD36C (17%). SMGs, including EGFR and RBM10, were associated with the dynamic evolution from AIS to IAC. Meanwhile, DEGs, including GPR143, CCR9, ADAMTS16, and others were associated with the entire process of invasive LUAD. We found that the signaling pathways related to cell migration and invasion were upregulated, and the signaling pathways of angiogenesis were downregulated across the pathological stages. Furthermore, we found that the messenger RNA (mRNA) levels of FAM83A, MAL2, DEPTOR, and others were significantly correlated with CNVs. Gene set enrichment analysis (GSEA) showed that heme metabolism and cholesterol homeostasis pathways were significantly upregulated in patients with EGFR/RBM10 co‐mutations, and these patients may have poorer overall survival than those with EGFR mutations. Based on the six calculation methods for the immune infiltration score, NK/CD8^+^ T cells decreased, and Treg/B cells increased with the progression of early LUAD.

**Conclusions:**

Our findings offer valuable insights into the unique genomic and molecular features of LUAD, facilitating the identification and advancement of precision medicine strategies targeting the invasive progression of LUAD from AIS to IAC.

## 1 INTRODUCTION

Early detection of lung cancer, as demonstrated in the National Lung Cancer Screening Trial,^1^ has the potential to greatly improve chances of successful treatment. The implementation of lung cancer screening protocols and the use of high‐resolution computed tomography (CT) for diagnostic imaging have led to a significant increase in the detection of pulmonary ground‐glass nodules (GGNs), among which a considerable portion represents preinvasive adenocarcinoma in situ (AIS), minimally invasive adenocarcinoma (MIA), and early‐stage invasive lung adenocarcinoma (IAC).^2^ AIS, the recognized precancerous state leading to IAC, is hypothesized to evolve into MIA and eventually into full IAC.^3^ As early‐stage lung adenocarcinomas manifest as ground‐glass nodules (GGNs), they serve as an excellent model for studying the initiation of lung carcinogenesis. However, the precise molecular delineation of these lesions remains largely unknown, and the evolutionary path from AIS to IAC remains a subject of ongoing debate. These aspects underscore the need for further research to elucidate the complex molecular landscapes and evolutionary dynamics involved in the progression of lung adenocarcinomas.

The molecular processes underlying the dynamic progression of lung cancer are yet to be fully understood, and have emerged as a prominent area of investigation among researchers in recent years. Many genetic factors, including mutations in epidermal growth factor receptor (EGFR), anaplastic lymphoma kinase (ALK), Kirsten rat sarcoma viral oncogene (KRAS), v‐Raf murine sarcoma viral oncogene homolog B (BRAF), mesenchymal‐epithelial transition gene (MET), and tumor protein p53 (TP53), are known to be involved in the development of LUAD.^4^, ^5^ Moreover, driver mutations, including those of EGFR, KRAS, and TP53, have been frequently identified in studies of LUAD,^6^, ^7^ but a significant number of LUAD patients have been found to lack these mutations.

To gain a better understanding of the molecular pathogenesis and underlying molecular determinants that contribute to the clinical behavior of early‐stage LUAD, we present a comprehensive description of the genomic and transcriptomic architecture of GGNs using whole‐exome sequencing (WES) and bulk RNA sequencing in 35 surgically resected cases of early‐stage LUAD. We aimed to determine whether mutational signatures, tumor heterogeneity, and specific altered signaling pathways could potentially elucidate the determinants of tumor behavior. This study will serve as compelling theoretical evidence to enhance clinical diagnosis and surgical intervention in early stage LUAD.

## 2 METHODS

### 2.1 Patient enrollment and specimen collection

The study protocol (#2020‐144‐01) was approved by the Ethics Committee of Xinqiao Hospital, Army Medical University, Chongqing, China. Informed consent was obtained from all patients before study initiation. Resected specimens were prospectively collected from patients at Xinqiao Hospital between May 2020 and November 2021 who presented with pulmonary GGNs and underwent resection. None of the patients received neoadjuvant chemotherapy or radiotherapy. Patients were eligible for inclusion based on the following criteria: (1) presence of pulmonary GGNs, with a maximum tumor diameter ≤3.0 cm; (2) histological diagnosis of lung adenocarcinoma (LUAD); (3) no prior history of other malignant tumors; (4) absence of regional lymph node metastasis based on pathological examination; (5) absence of distant metastasis; and (6) availability of sufficient specimens of pulmonary adenocarcinoma and adjacent normal tissue for WES and RNA‐Seq analysis. Pathological assessments were independently conducted by three experienced pathologists following the fifth edition of the WHO lung tumor classification system.

Tumors and matched normal adjacent tissues were collected with informed consent for WES and RNA‐Seq analysis. Each sample was obtained with a minimum size of 0.3 cm. The samples were rapidly frozen in liquid nitrogen and stored at −80°C in a refrigerator in the operating room to preserve the integrity of DNA and RNA.

### 2.2 DNA extraction and library preparation

Genomic DNA was extracted using the cetyltrimethylammonium bromide (CTAB) method, following the manufacturer's instructions. DNA quantification was performed using the Nanodrop 2000 spectrophotometer (Thermo Fisher Scientific, Inc., Wilmington, DE), while 1% agarose gel electrophoresis was used to assess DNA integrity. Agilent SureSelect Human All Exon v8 library (Agilent Technologies) was used to capture genomic DNA samples as per the manufacturer's instructions. Briefly, the genomic DNA was sheared into short fragments, followed by ligation of adapters to the polished ends of the DNA fragments. The libraries were amplified using polymerase chain reaction (PCR). Hybridization of the amplified libraries to the probes was carried out, followed by washing and elution of the DNA fragments bound to the probes using the kit‐supplied buffer. The libraries were sequenced using an Illumina sequencing platform (NovaSeq 6000, Illumina, Inc., San Diego, CA), generating 150 bp paired‐end reads.

### 2.3 RNA extraction and library preparation

Total RNA was extracted using the TRIzol reagent (Invitrogen, Carlsbad, CA) following the provided protocol by the manufacturer. RNA quality was assessed using an Agilent 2100 Bioanalyzer (Agilent Technologies), and all samples exhibited RNA integrity values greater than 6.0. RNA‐Seq libraries were prepared using the VAHTS Universal V6 RNA‐Seq Library Prep Kit as per the manufacturer's guidelines. In summary, the mRNA was purified using oligo (dT) magnetic beads The mRNA was further fragmented shorter fragments using a fragmentation buffer. First‐strand cDNA synthesis was performed using random hexamer primers and the fragmented mRNA as templates. A mixture of buffer, dNTPs, RNase H, and DNA polymerase I was subsequently added to synthesize the second strand. The double‐stranded cDNAs were purified and eluted using EB buffer, followed by end repair and the addition of poly(A). Subsequently, sequencing adapters were ligated to the 5′ and 3′ ends of the fragments. The fragments were then purified and underwent PCR amplification to create a cDNA library. The libraries were sequenced on an Illumina Novaseq 6000 platform, generating 150 bp paired‐end reads.

### 2.4 WES

WES and analysis were performed by OE Biotech Co. Ltd. in Shanghai, China. The Illumina NovaSeq 6000 machine was used to obtain paired‐end sequence data, with an average sequencing depth of 133.6X for normal tissues and 146.8X for tumor tissues. Alignment of the sequence data to the human reference genome (NCBI, GRCh37.p13) was carried out using the Burrows–Wheeler Aligner (BWA) software, version 0.7.17. PCR duplicates were then sorted and removed using Sambamba. For recalibration of the base quality score and realignment of single nucleotide polymorphisms and insertion/deletion (INDELs), GATK, version 4.1.9.0, was employed.

### 2.5 RNA‐sequencing

The transcriptome sequencing and analysis were performed by OE Biotech Co., Ltd. in Shanghai, China. For each sample, approximately 49.2 million raw reads were generated. These raw reads, in fastq format, underwent initial processing using fastp,^8^ which included the filtration of low‐quality reads to obtain clean reads. After this filtration step, around 48.5 million clean reads were retained for further analyses. Subsequently, the clean reads were aligned to NCBI GRCh38.p13 using HISAT2,^9^ and the expression of each gene was determined utilizing HTSeq count.^10^

### 2.6 Western blot

For western blotting, tissues were lysed in lysis buffer. Supernatant was collected by centrifugation and separated by SDS‐PAGE. After transfer, polyvinylidene difluoride (PVDF) filters were incubated with 1:1000 anti‐MAL2 (bs‐7175R, Bioss antibodies, Beijing, China), DEPTOR (D263703, Sangon Biotech, Shanghai, China), SRD5A1(26001‐1‐AP, Proteintech, Chicago, USA), LAPTM4B (A10761, ABclonal, Wuhan, China), β‐actin (4967S, Cell Signaling Technology, Boston, USA) followed by 1:4000 rabbit anti‐goat HRP‐conjugated secondary antibody (ZB‐2306, ZSGB, Beijing, China), and developed using the Western BLoT Chemiluminescence HRP Substrate (T7101Q, Takara Biomedical Technology, Beijing, China).

### 2.7 Data analysis

The WES analysis was centered on various types of variants, including single‐nucleotide variants (SNVs), insertion and deletion (InDel) mutations, copy number variations (CNVs), mutation spectrum, tumor mutation burden (TMB), and high‐frequency gene mutations. Somatic SNVs and InDel analyses were conducted using Mutect2 within GATK4 (Version: 4.1.9.0) software. To detect somatic CNVs in lung tumor samples and their corresponding normal lung samples, Control‐FREEC (Version: 11.3) software was employed with default settings. The mutations detected in the normal tissues of patients were compared with the Cancer Gene Census (CGC) database and the Rahman database^11^ to screen for possible cancer susceptibility genes. The mutation features of somatic SNV were extracted by the method of Nonnegative Matrix Factorization (NMF).^12^

For RNA‐Seq analysis, the Gene Set Enrichment Analysis (GSEA) was conducted to identify differentially expressed genes (DEGs) based on a false discovery rate (FDR) < 0.05 and a fold‐change >2 using DESeq2.^13^ Subsequently, enrichment analyses for DEGs were performed using gene ontology (GO), Kyoto Encyclopedia of Genes and Genomes (KEGG) pathway, and Reactome through hypergeometric distribution. The bubble diagram depicting significant enrichment terms was generated using R (v 3.2.0). Interactions between genes were determined using the STRING database (https://cn.string‐db.org/, version 12.0) and PPI networks were visualized in Cytoscape (version 3.9.0). GSEA utilizes predefined gene sets, including Hallmark gene sets (h.all.v7.2.symbols.gmt), GO gene sets (c5.all.v7.2.symbols.gmt), and curated gene sets from KEGG (c2.cp.kegg.v7.2.symbols.gmt). Enrichment within the sorted list of differentially expressed genes is assessed based on rankings, where enrichment toward the top indicates upregulation and toward the bottom indicates downregulation. The analysis incorporates various statistics, such as the enrichment score (ES), which quantifies the level of enrichment, and the normalized enrichment score (NES), which normalizes the ES according to gene set size. Additionally, false discovery rate (FDR) estimates the probability of false‐positive discoveries, while the nominal p value indicates the statistical significance of the ES derived from a functional gene set. A leading‐edge analysis was also performed to identify overlapping genes between enriched functional gene sets obtained from the previous analysis.

### 2.8 Connectivity Map (Cmap) analysis

Differentially expressed genes between patients carrying EGFR mutations and EGFR/RBM10 double mutations were obtained with mRNA expression data (Transcripts Per Million, TPM) from patients of our hospital and the TCGA‐LUAD dataset using the R package limma v3.46.0. The top 250 upregulated and downregulated genes were collected and used as the EGFR/RBM10 double mutation‐related signatures. CMap analysis was conducted as previously described.^14^, ^15^

### 2.9 Immunoinfiltration analysis

The immunoinfiltration analysis was carried out by R‐packet IOBR, and the relative quantification of the cell microenvironment of tumor tissue was performed by deconvolution algorithm. IOBR integrated eight tumor microenvironment quantization methods, including CIBERSORT, TIMER, xCell, MCPcounter, ESITMATE, EPIC, IPS, quantTIseq, and collected 255 published feature gene sets to achieve effective evaluation of tumor tissue microenvironment.

### 2.10 Statistical analysis

SPSS software (version 25.0; IBM Corp., Armonk, NY) was used for all statistical analyses. Analysis of variance was employed for normally distributed continuous data, while the Wilcoxon test was used for non‐normally distributed continuous data. Student's *t* test was utilized for continuous variables with a normal distribution. The chi‐squared test (R × C) was used for categorical variables analysis. Statistical significance was set at *p* < 0.05.

## 3 RESULTS

### 3.1 Clinical information of GGN patients enrolled for sequencing

**FIGURE 1:**
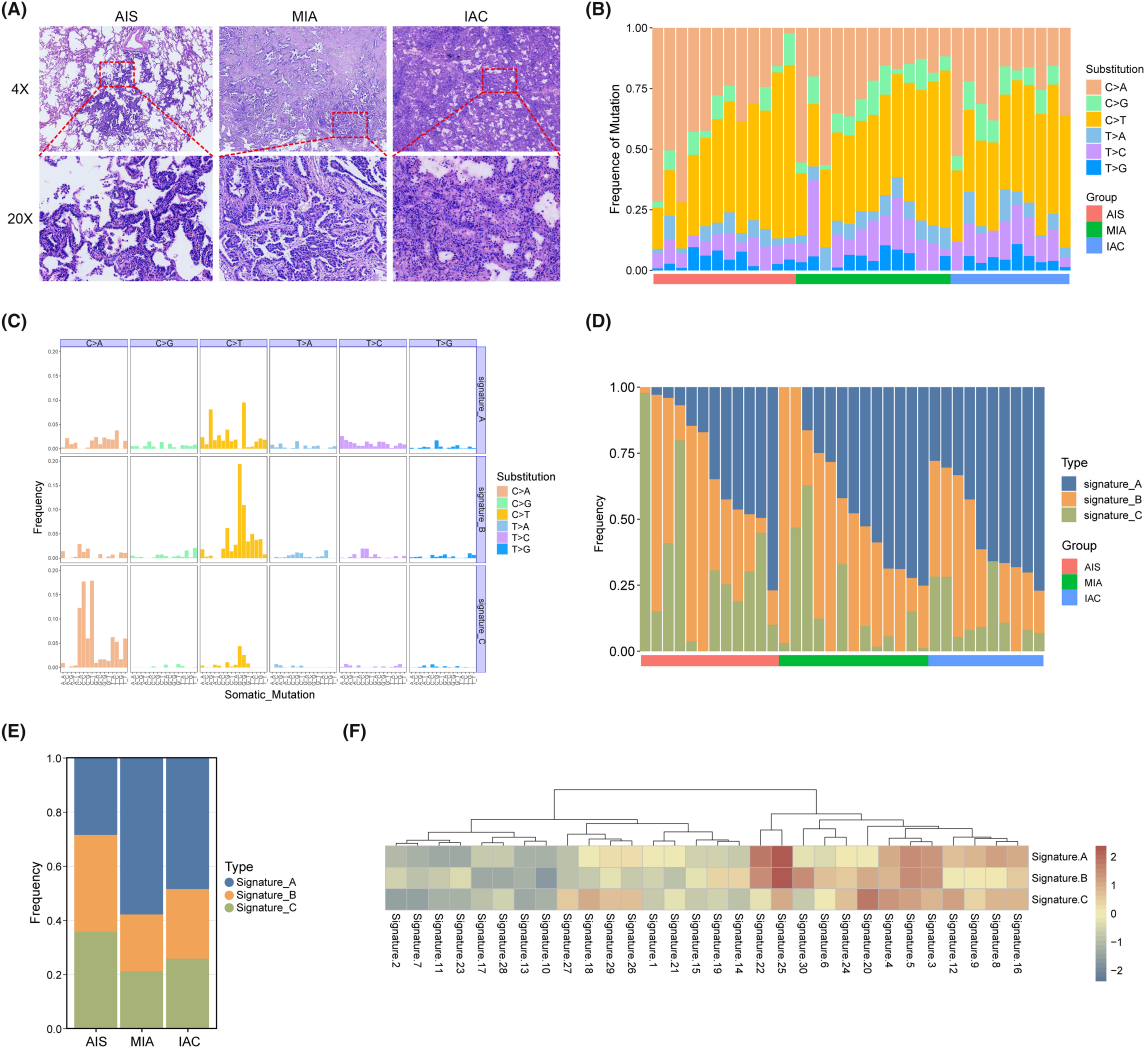
Mutational signature in LUAD of different histologic subtypes. (A) Representative IHC images for AIS, MIA, and IAC tissues. (B) Relative contribution of the indicated mutation types to the point mutation spectrum for each tissue type. (C) Relative contribution of each indicated trinucleotide change to the three mutational signatures that were identified by NMF analysis of the somatic mutation catalogs. (D) Relative contribution of each mutational signature for each tissue type. (E) Statistical chart of each mutational signature for each tissue type. (F) Heatmap showing the cosine similarity of the mutational signature with the COSMIC signatures.

A total of 70 samples were collected from 35 patients with pulmonary GGNs, including 12 AIS, 13 MIA, 10 IAC, and 35 matched normal adjacent tissues. Whole‐exome and bulk RNA sequencing were performed on all tumors and matched normal adjacent tissues. The diagnosis of LUAD was confirmed through a histological review conducted by three experienced pathologists, using hematoxylin and eosin staining and immunohistochemistry (IHC) (Figure 1A). The cohort consisted of 18 patients with pGGN and 17 patients with mGGN. Among the 35 patients, there were 27 females and 8 males, with a median age of 55. Detailed clinical information can be found in Table 1. The statistical analysis results indicate that the pathological subtypes are significantly correlated with GGO type and nodule size, but not with gender, smoking history, and nodule location.

**TABLE 1:** Clinicopathological features of early lung cancers with different pathological stages.

Variables	Pathological subtypes			*p* value
	AIS (*n* = 12)	MIA (*n* = 13)	IAC (*n* = 10)	
Age (years)	49.58 ± 14.41	54.54 ± 13.23	58.90 ± 7.23	
Gender				
Male	2	4	2	
Female	10	9	8	0.819^a^
Smoking history				
Never	11	12	8	
Ever	1	1	2	0.416^a^
GGO components				
Pure GGO	12	6	0	
Mixed GGO	0	7	10	0.000^b^
Tumor size (mm)				
<10	9	5	1	
10–20	3	8	3	
>20	0	0	6	0.000^a^
Position of nodule				
RUL	2	5	4	
RML	2	3	0	
RLL	0	2	3	
LUL	4	3	2	
LLL	4	0	1	0.135^a^

^a^ Linear‐by‐linear association.

^b^ Pearson chi‐Square.

### 3.2 Characterization of mutational signatures in GGN progression

The WES analysis encompassed a total of 35 tumor samples along with their corresponding adjacent normal tissues. Among the tumor samples, the average sequencing depth reached 146.8×, ensuring a minimum depth of 10× coverage in 98.53% of the target exome regions. In contrast, the normal tissues exhibited an average sequencing depth of 133.6×, with at least 10× coverage in 98.46% of the target exome regions.

Overall, the mutation spectrum was similar among the different histological subtypes, and the mutational spectrum of the six substitutions showed that C > T or C > A transversions were dominant in most GGNs (Figure 1B). Next, we used the NMF method to decipher mutational signatures of different histological subtypes. Three prominent signatures were identified and compared with known COSMIC signatures using cosine similarity. As shown in Figure 1C, signature A and B had more frequent C > T mutations than signature C, at the same time, it was found that signature C exhibited a higher frequency of C > T mutations. We also compared the frequency of signatures in each histological subtypes and found that there were certain changes accompany with pathological escalation, especially for signature A (Figure 1D,E). As shown in Figure 1F, the three signatures had similar biological functions. Signatures A and B correlated strongly with both COSMIC signatures 25 (cosine similarity 0.62 and 0.46) and 22 (cosine similarity 0.51 and 0.36), and signature C also had a certain correlation with Signature 25 (cosine similarity 0.42). This indicates that biological functions linked with Signature 25 may play a vital role in increasing the malignancy grade of early LUAD; however, the etiology of Signature 25 is currently unknown. The molecular mechanisms underlying signatures A, B, and C are unknown; however, both signatures have been reported to have a transcriptional strand bias. In contrast, Signature C had a spiky C > A mutation, resembling COSMIC Signature 20 (cosine similarity: 0.34). Exited evidence has shown that COSMIC Signature 20 is associated with defective DNA mismatch repair, and therefore may have an impact on transcriptional regulation.^16^

### 3.3 Identification of somatic mutations and their roles in GGN progression

**FIGURE 2:**
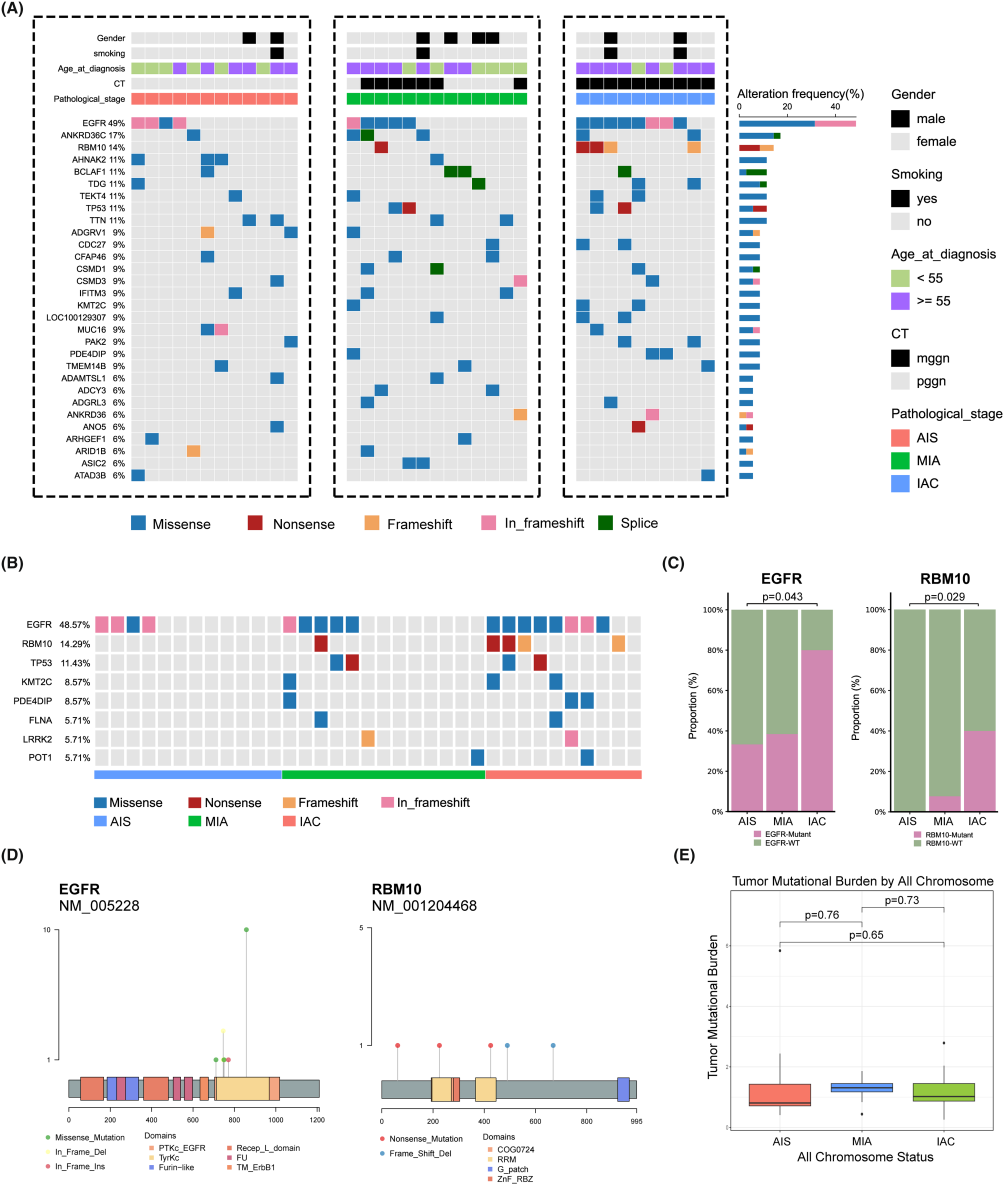
Overview of the LUAD data and mutational landscape. (A) Mutations with high somatic mutational frequencies in LUAD of different histologic subtypes. (B) Mutations with high driver gene frequencies. (C) The proportion of EGFR and RBM10 gene mutation in different histologic subtypes. (D) Hot spots of mutations in the EGFR and RBM10 gene. (E) TMB in different histologic subtypes.

A total of 5146 somatic SNVs and 471 indels were identified in all tumor samples. We found frequent somatic mutations in EGFR (49%, 17/35), ANKRD36C (17%, 6/35), RBM10 (14%, 5/35), TP53 (11%, 4/35), AHNAK2 (11%, 4/35), BCLAF1 (11%, 4/35), TDG (11%, 4/35), TEKT4 (11%, 4/35), and TTN (11%, 4/35). As shown in Figure 2B, EGFR was the most mutated cancer gene, occurring in four of the twelve AISs and eight of the ten IACs (*p* = 0.043). In addition, RBM10 was detected in zero of twelve AISs but in four of ten IACs (*p* = 0.029). There was a significant difference in the incidence of cancer gene mutations between the different pathological subtypes (Figure 2C). These results suggest that EGFR and RBM10 mutations play important roles in the carcinogenesis of LUAD. Next, we analyzed the EGFR and RBM10 mutation sites in these patients. The most frequent EGFR mutation was p.L858R, which was found in ten of seventeen patients (Figure 2D). In addition, there was no significant difference in the TMB among the three tumor subtypes (Figure 2E).

**FIGURE 3:**
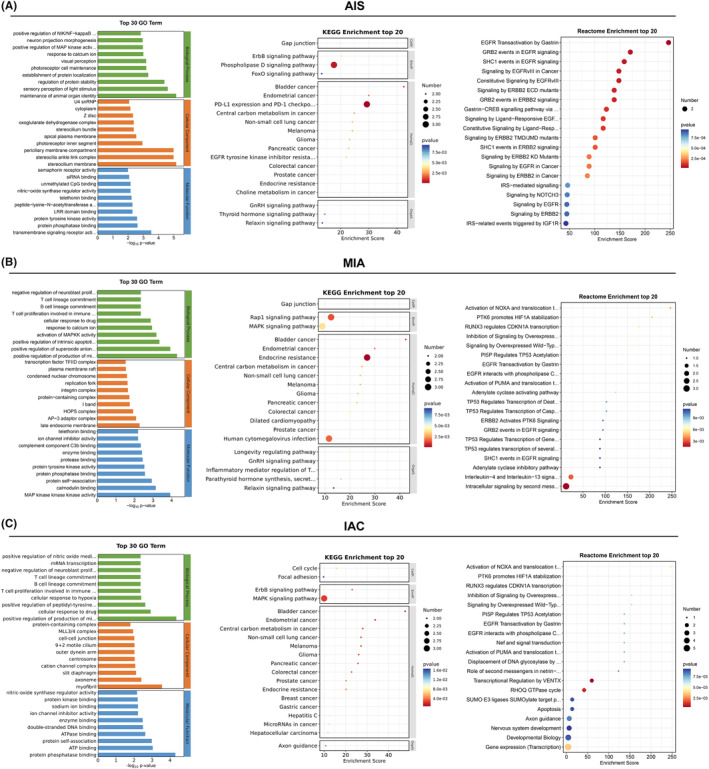
Enrichment analysis related to SMGs. (A) Results of GO, KEGG, and Reactome enrichment of SMGs in AIS. (B) Results of GO, KEGG, and Reactome enrichment of SMGs in MIA. (C) Results of GO, KEGG, and reactome enrichment of SMGs in IAC.

Focusing on the alteration of functions and pathways in cancer, SMGs in all pathological subtypes were evaluated using enrichment analysis. Based on the Go enrichment analysis results, AIS exhibits completely different biological processes compared to MIA and IAC. AIS is primarily enriched in positive regulation of NIK/NF‐KB, positive regulation of MAP kinase activity, photoreceptor of protein localization, and positive regulation of protein stability, while MIA and IAC have similar biological processes, mainly enriched in the T cell lineage commitment, B cell lineage commitment, T cell proliferation involved in immune response, and positive regulation of production of miRNAs. This result suggests that AIS exhibits entirely different biological behaviors before and after (Figure 3A–C). KEGG enrichment analysis showed that SMGs of AIS were significantly enriched in gap junction, phospholipase D signaling pathway and ErbB signaling pathway, while MIA and IAC were significantly enriched in focal adhesion and MAPK signaling pathway. Reactome enrichment analysis showed that SMGs of MIA and IAC have different enrichment signaling pathway compared to AIS. AIS is primarily enriched in EGFR transactivation by gastrin, GRB2 events in EGFR signaling, SHC1 events in EGFR signaling, and signaling by EGFRvIII in cancer, while MIA and IAC mainly enriched in the activation of NOXA and translocation to mitochondria, PTK6 promotes HIF1A stabilization, RUNX3 regulates CDKN1A transcription, and inhibition of signaling by overexpress.

### 3.4 RNA sequencing and gene expression profiling

#### 3.4.1 Transcriptomic differences between tumor and normal tissues

**FIGURE 4:**
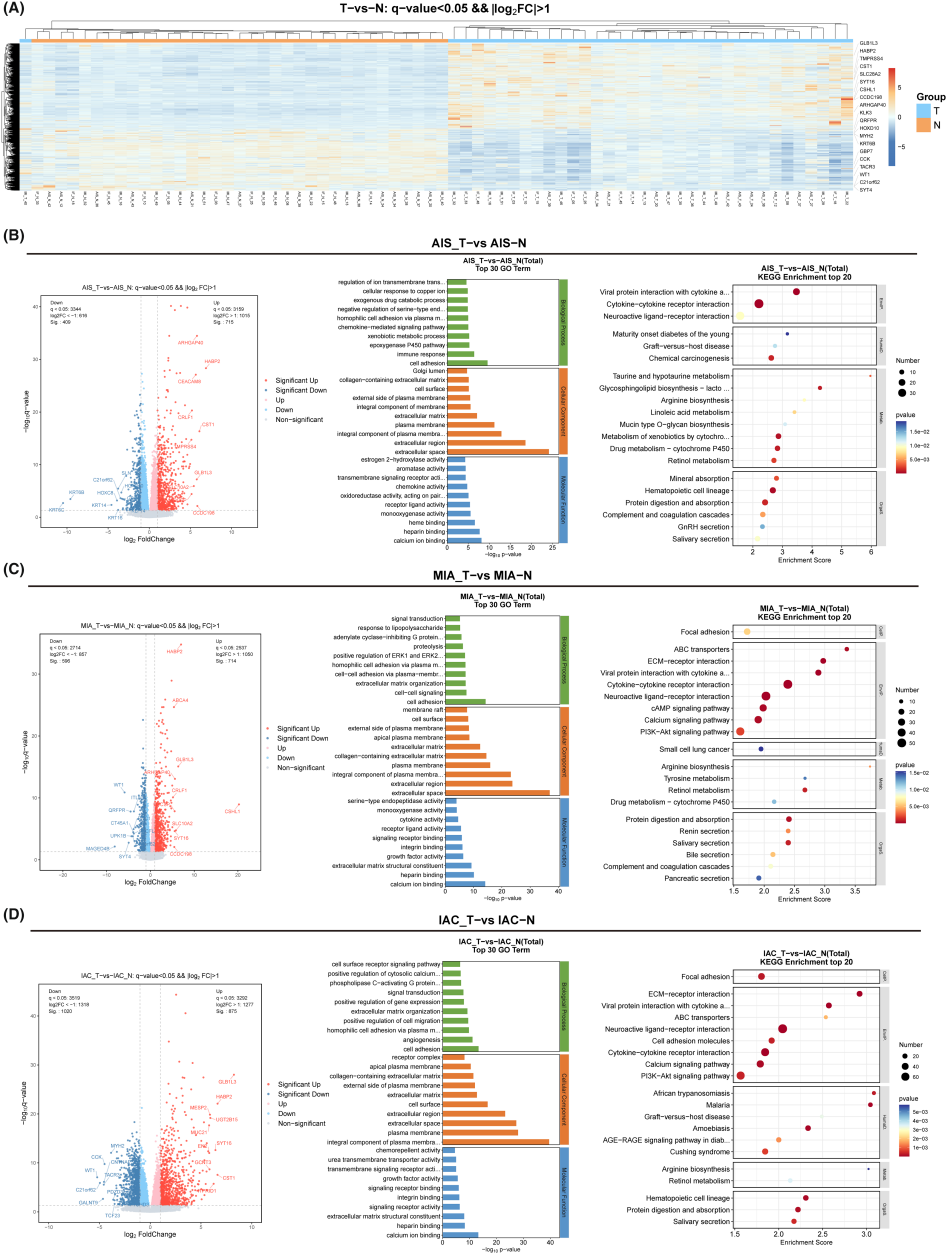
The transcriptomic landscape between tumor and normal tissues. (A) Heatmap of differentially expressed genes (DEGs) with log2 fold‐change >1 and *p* < 0.05. Hierarchical clustering analysis was performed on DEGs identified in tumor and normal tissues. Expression levels are represented by color, where blue indicates low expression and pink‐orange indicates high expression. (B) Volcano plot of DEGs with log2 fold‐change >1 and *p* < 0.05, and enrichment results of DEGs in AIS. (C) Volcano plot of DEGs with log2 fold‐change >1 and *p* < 0.05, and enrichment results of DEGs in MIA. (D) Volcano plot of DEGs with log2 fold‐change >1 and *p* < 0.05, and enrichment results of DEGs in IAC.

To assess the differential expression levels between tumor and normal tissues, the relative gene expression levels were categorized into upregulated and downregulated genes. Hierarchical clustering analysis unveiled a shared set of 1503 DEGs between the tumor and normal tissues. Among these, 864 exhibited upregulation, while 639 displayed downregulation (Figure 4A). These results showed that the expression levels of GLB1L3, HABP2, TMPRSS4, CST1, SLC28A2, SYT16, CSHL1, CCDC198, and ARHGAP40 were only significantly increased in tumor tissues compared to normal tissues. The levels of SYT4, WT1, TACR3, CCK, GBP7, KRT6B, MYH2, HOXD10, and QRFPR were reduced in tumor tissues. Next, we used a volcano plot to further observe the DEGs between each pathological subtype and normal tissue. Our results showed that there are certain differences in upregulated and downregulated genes in the three pathological subtypes. Specifically, in AIS, the significantly upregulated genes were ARHGAP40, HABP2, CEACAM8, CRLF1, and CST1 (Figure 4B), while in MIA, the significantly upregulated genes were HABP2, ABCA4, GLB1L3, and ARHGAP40 (Figure 4C), and in IAC, the significantly upregulated genes were GLB1L3, HABP2, MESP2, MUC21, and UGT2815 (Figure 4D). Due to the differences in gene expression between pathological subtypes, subsequent functional enrichment analysis also showed different results. Specifically, AIS was primarily enriched in cell adhesion, immune response, calcium ion binding, viral protein interaction with cytokine, and cytokine–cytokine receptor interaction (Figure 4B); MIA was primarily enriched in cell adhesion, cell–cell signaling, extracellular space, ABC transporters, and EMC–receptor interaction (Figure 4C); and IAC was primarily enriched in cell adhesion, angiogenesis, and integral component of plasma membrane (Figure 4D).

#### 3.4.2 Transcriptomic differences between three pathological subtypes

**FIGURE 5:**
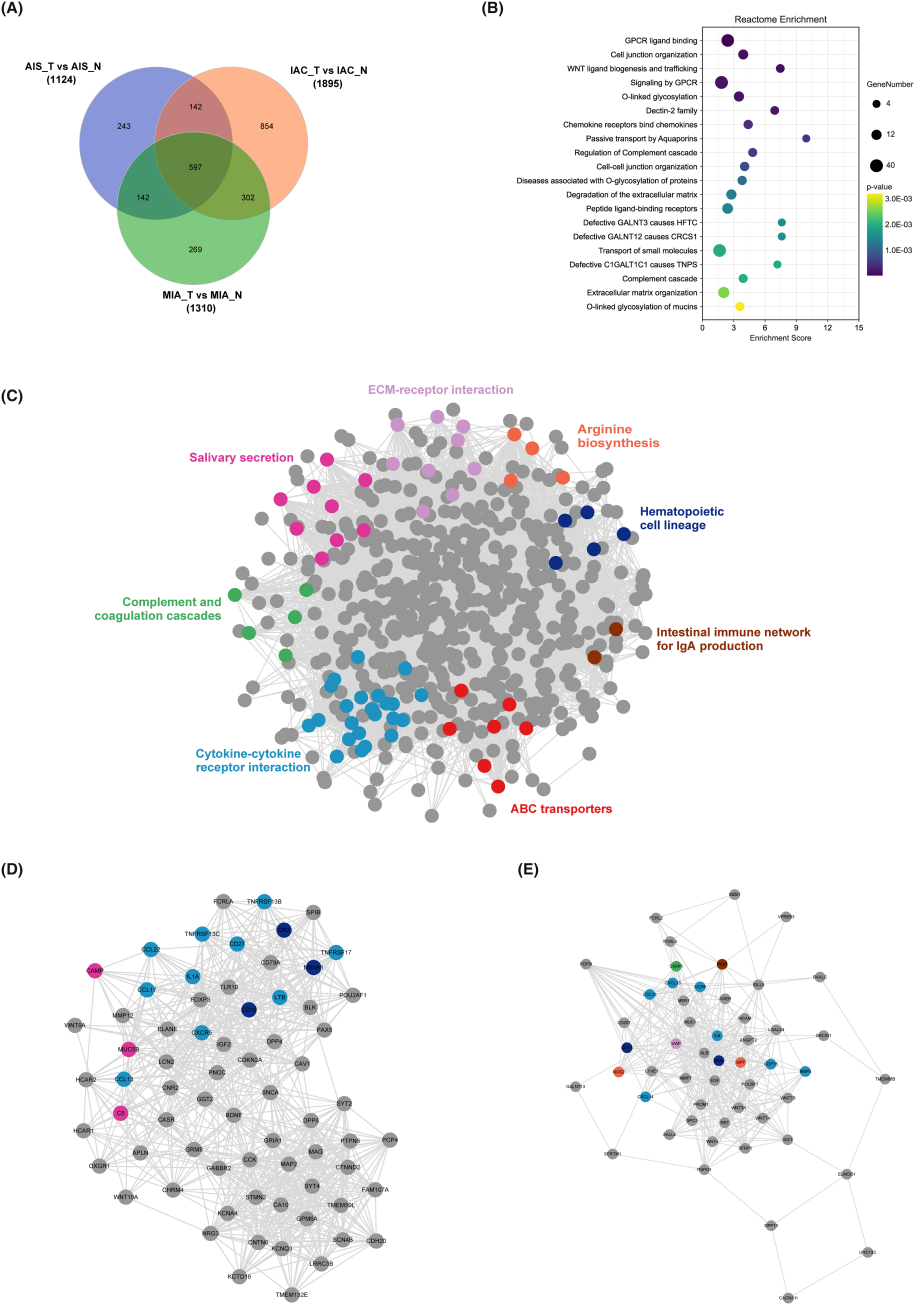
Comparison of DEGs concordance of pathological subtypes. (A) Venn diagrams of DEGs between pathological subtypes. (B) Reactome enrichment analysis of between pathological subtypes. (C) The protein–protein interaction (PPI) network of the shared DEGs. (D, E) The results of the MCODE plugin clustering analysis. The nodes represent proteins, and the edges represent interactions.

To gain molecular insight into the differences between pathological subtypes, we used the R packages edgeR and DEseq to identify differentially expressed genes (DEGs). We found a total of 1124 DEGs in AIS, 1310 DEGs in MIA, and 1895 DEGs in IAC, with |log2FoldChange| > 1 and FDR < 0.01 for both methods. Among these DEGs, 597 were shared among all three pathological subtypes (Figure 5A). We conducted Reactome enrichment analysis on the shared DEGs, which revealed differences in signaling pathways between tumor and normal tissues (Figure 5B). Specifically, the shared DEGs were enriched in well‐known carcinogenesis pathways such as “GPCR ligand binding,” “cell junction organization,” “WNT ligand biogenesis and trafficking,” “signaling by GPCR,” and “O‐linked glycosylation.”

For demonstrating potential protein interaction (PPI) correlations, the PPI network of the shared DEGs was constructed using STRING and visualized using Cytoscape software. There were 576 nodes and 8957 edges in the PPI network. Different colors indicate the diverse pathways in which the genes participate. Among these, key signaling pathways were identified from the network of shared DEGs using the MCODE plugin, including ECM–receptor interaction, salivary secretion, complement and coagulation cascades, cytokine–cytokine interaction, ABC transporters, intestinal immune network for IgA production, hematopoietic cell lineage, and arginine biosynthesis (Figure 5C). Among them, ECM–receptor interaction, cytokine‐–cytokine interaction are well‐known signaling pathways for the progression of different stages of lung cancer.^17^, ^18^ Then, using degree ≥10 as the cutoff value, two clusters with high scores were identified (Figure 5D) and are listed in Table 2. Interestingly, proteins involved in cytokine and cytokine receptor interactions were all found in both core protein–protein interaction regulatory modules, suggesting that during the malignant progression of nodules, cytokine‐mediated signaling pathways may be abnormally activated.

**TABLE 2:** Cluster details based on MCODE plug‐in.

Clusters	Targets contained in clusters	Score	Nodes	Edges
1	PNOC, CNTN6, GABBR2, C5, MAP2, POU2AF1, SYT4, STMN2, LRRC3B, CASR, NRG3, PAX5, IGF2, MAG, FCRLA, SCN4B, GPM6A, TNFRSF13B, DPP4, CAV1, CXCR5, CCK, CR2, GRIA1, CD79A, CD19, HCAR2, GRM8, CHRM4, TLR10, PCP4, APLN, LCN2, TMEM59L, BDNF, KCNA4, DPP6, KCNQ3, CDH20, IL1A, TNFRSF17, TMEM132E, CD27, CDKN2A, CA10, MUC5B, SYT2, CCL13, FAM107A, ELANE, CAMP, WNT9A, GGT2, SPIB, OXGR1, HCAR1, WNT10A, CTNND2, TNFRSF13C, BLK, MS4A1, LTB, SNCA, CCL17, FOXP3, PTPN5, MMP12, KCTD16, CCL22, and CNR2	22.638	70	781
2	IL6, PROM1, C5AR1, CACNA1I, FCRL2, BAALC, MMP7, ALB, PIGR, CD207, ELMOD1, MZB1, CD1A, SIX1, CCL19, NOS2, DPP10, IGLL5, SALL4, POU5F1, MME, LGALS4, CXCL13, VWF, MCAM, ANGPT2, GPC3, RET, WNT7A, SERTM1, CCR6, TMEM88B, WNT7B, WNT3A, WNT4, UNC13C, SFRP1, AGER, MUC1, SRCIN1, PDPN, LYVE1, CXCL14, VPREB3, EGF, GALNT13, GPT, RSPO1, GDF15, FCRL5, MSR1, and BMP3	15.02	52	383

**FIGURE 6:**
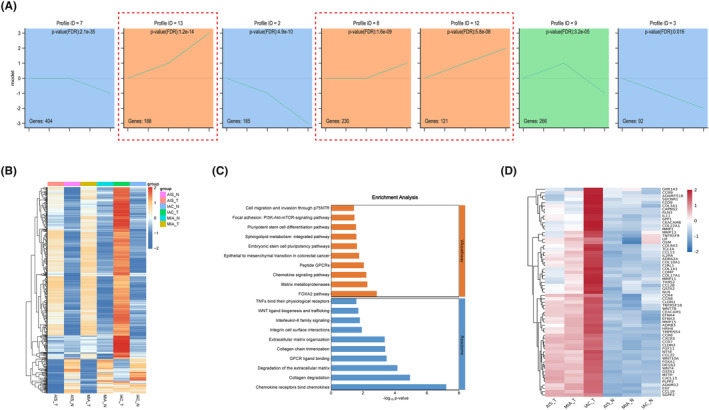
Upregulated DEGs and associated biological progresses in malignant transformation. (A) Trend chart of DEGs by STEM analysis. (B) Heatmap of upregulated DEGs between tumor and normal tissue in different histologic subtypes. (C) Wikipathway and reactome enrichment analysis of upregulated DEGs. (D) Heatmap of upregulated DEGs with a gradual ascent.

To gain a deeper understanding of the dynamic mechanisms underlying LUAD progression, we performed STEM analysis to elucidate the trends in DEGs among the AIS, MIA, and IAC subgroups. The results illustrate the prominently enriched genetic alteration trends derived from STEM trend analysis. Among these trends, the three orange‐colored trends signified a gradual increase across AIS, MIA, and IAC, encompassing 539 genes (Figure 6A). A heatmap was generated for the 539 genes, illustrating the comparison between tumor and normal samples (Figure 6B). The results showed the expression patterns of these genes across pathological subtypes. The findings revealed that the majority of genes were significantly upregulated in the tumor samples, with a gradual increase observed across AIS, MIA, and IAC. However, it is noteworthy that a subset of genes, while showing progressive upregulation in tumor samples from AIS, MIA, and IAC, was downregulated in the tumor samples compared to the normal samples. After excluding a subset of genes that were downregulated in tumor tissues, 466 genes remained. These genes, which exhibited a progressive increase across pathological stages, may represent key genes involved in the dynamic evolution of LUAD. Reactome and Wikipathway enrichment analyses showed that the key genes were significantly enriched in cell migration and invasion through the p75NTR, PI3K‐Akt‐mTOR signaling pathway, pluripotent stem cell differentiation pathway, FOXA2 pathway, chemokine signaling pathway, collagen degradation, and chemokine receptor‐binding chemokines (Figure 6C). For genes within the pathways depicted in the bar graph, the selection process focused on genes that demonstrated concentrated functional enrichment and significance. After consolidation, 63 genes exhibiting a gradual increase across AIS, MIA, and IAC were identified, underscoring their importance in the dynamic evolution from AIS to IAC (Figure 6D).

### 3.5 Insights from CNV‐mRNA correlations in GGN progression

**FIGURE 7:**
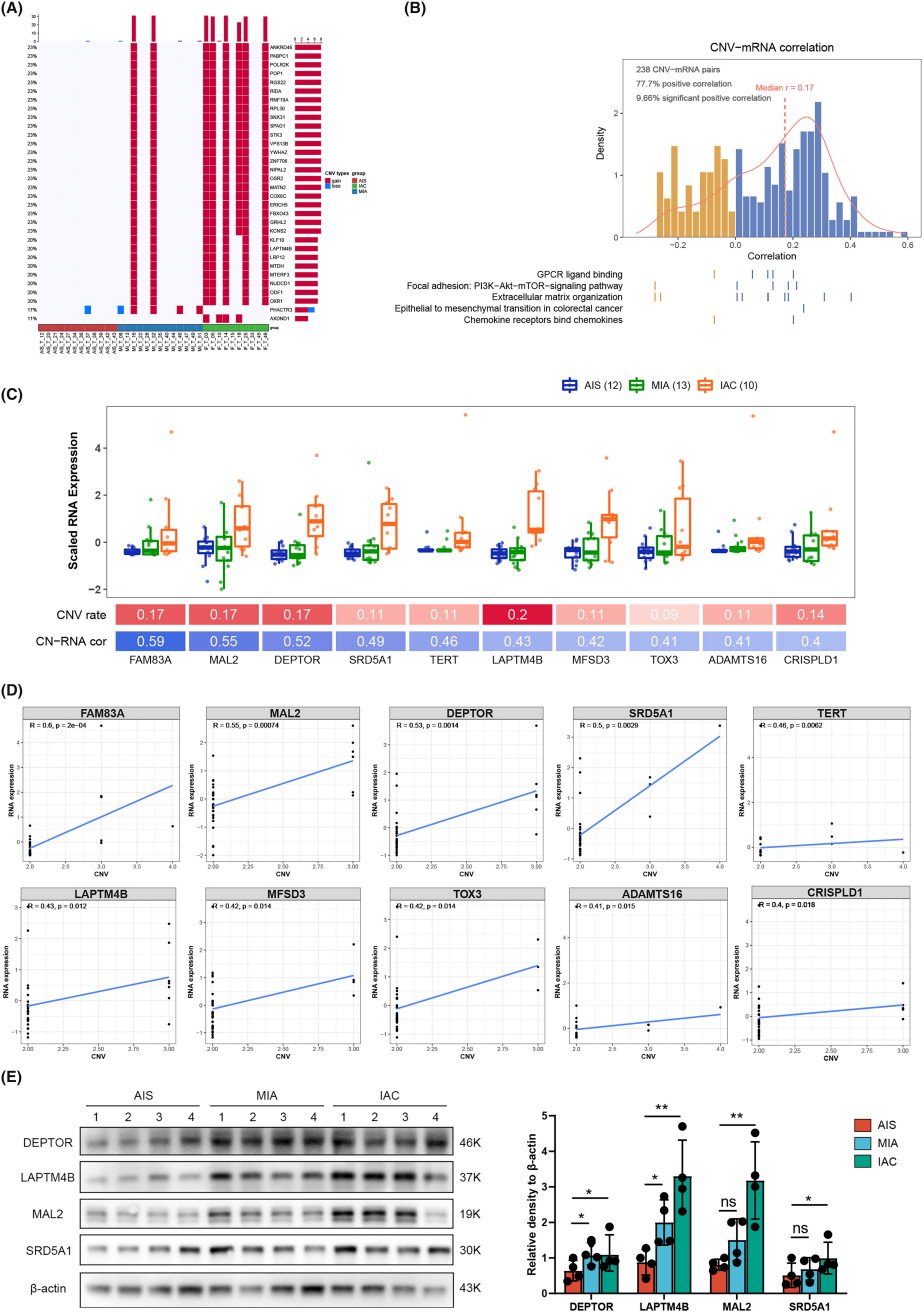
Correlation of mRNA expression with CNV status. (A) Overview of the top 30 genes with CNVs. (B) Correlation between mRNA expression and CNVs. (C) The top ten genes with the highest correlation scores between mRNA and CNVs. (D) Scatter diagram of the correlation between mRNA and CNVs. (E) Western blotting results for top 4 proteins in tumor tissues of twelve patients with different pathological stages.

Large‐scale CNVs were observed in the three tumor subtypes compared to normal tissues. These genes, including ANKRD46, PABPC1, POLR2K, POP1, RGS22, RIDA, RNF19A, RPL30, SNX31, SPAG1, and STK3, were significantly amplified and gradually increased from AIS to IAC (Figure 7A). To further explore the mechanism underlying the invasive process of LUAD at the molecular level, we integrated our analysis of DEGs and CNVs. Of the 466 genes that were progressively upregulated in the pathological subtypes, 238 showed a significant correlation between copy number and the corresponding RNA levels. In the CNV‐mRNA pairs, 77.7% had positive Spearman correlations with GPCR ligand binding, the PI3K‐Akt‐mTOR signaling pathway, and extracellular matrix organization positively correlated in tumors (Figure 7B). Furthermore, we found that the mRNA expression levels of FAM83A, MAL2, DEPTOR, SRD5A1, TERT, LAPTM4B, MFSD3, TOX3, ADAMTS16, CRISPLD1, and AFF3 were significantly correlated with CNVs (Figure 7C,D). To confirm the expression of the significantly upregulated genes in patients' tumor tissues, we conducted Western Blot (WB) experiments. The results revealed a notable increase in gene expression with the continuous progression of the pathology (Figure 7E).

### 3.6 Comparison between patients with EGFR/RBM10 co‐mutation and EGFR mutation

**FIGURE 8:**
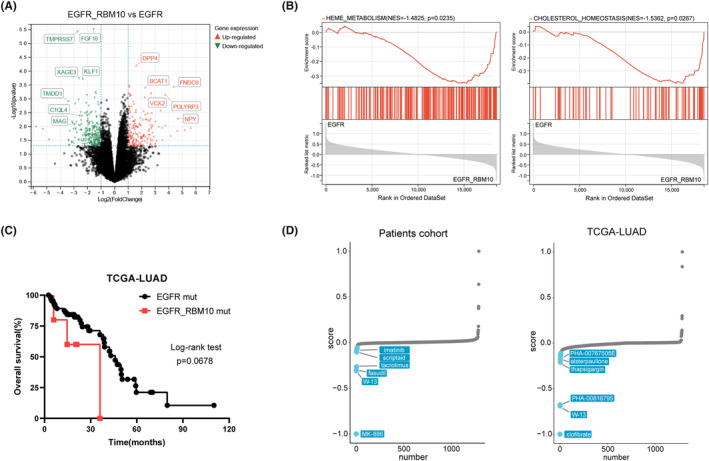
Correlation of mRNA expression with EGFR/RBM10 co‐mutations. (A) Volcano plot of DEGs between EGFR/RBM10 co‐mutations and EGFR mutations only. (B) GSEA enrichment analysis of DEGs. (C) The overall survival rates between EGFR/RBM10 co‐mutations and EGFR mutation only by TCGA database. (D) Results of CMap analysis using eXtreme Sum method. The top ranked ten compounds with highest reversal potency are illustrated in the right panel.

We observed that RBM10 mutations were frequently associated with EGFR mutations. To further understand the mechanism of lung cancer mutations, we divided 17 patients with EGFR mutations into two groups: those with EGFR/RBM10 co‐mutations and those with only EGFR mutations. We compared the mRNA expression profiles of the two groups and found that the mRNA expression levels of DPP4, BCAT1, FNDC9, VCX2, PGLYRP3, and NPY were significantly upregulated in the EGFR/RBM10 co‐mutation group (Figure 8A). GSEA demonstrated that the co‐mutation group was enriched in pathways including heme metabolism (enrichment score [NES] = −1.485, *p* = 0.0235) and cholesterol homeostasis (NES = −1.5362, *p* = 0.0267) signaling (Figure 8B). We compared the overall survival rates between the two groups using clinical data from the TCGA database. The results indicated that the co‐mutation group showed slightly poorer survival than the group with only EGFR mutations; however, this difference was not statistically significant (Figure 8C). Furthermore, we used CMap analysis to explore potential drugs that could target lung cancer with EGFR/RBM10 co‐mutations. The top 500 differentially expressed genes from our own patient cohort and the TCGA‐LUAD database were analyzed, respectively, and the EGFR/RBM10 co‐mutation‐related gene signature was matched with CMap gene signatures by using eXtreme Sum (XSum) method. The Xsum score for each compound is shown in Figure 8D. W13, a potent MsbA inhibitor,^19^ simultaneously appeared in the results of both datasets with an obviously low score, indicating that W13 may have an inhibitory effect on lung cancer cells carrying EGFR/RBM10 co‐mutations.

### 3.7 Immune cell microenvironment of different pathological subtypes

**FIGURE 9:**
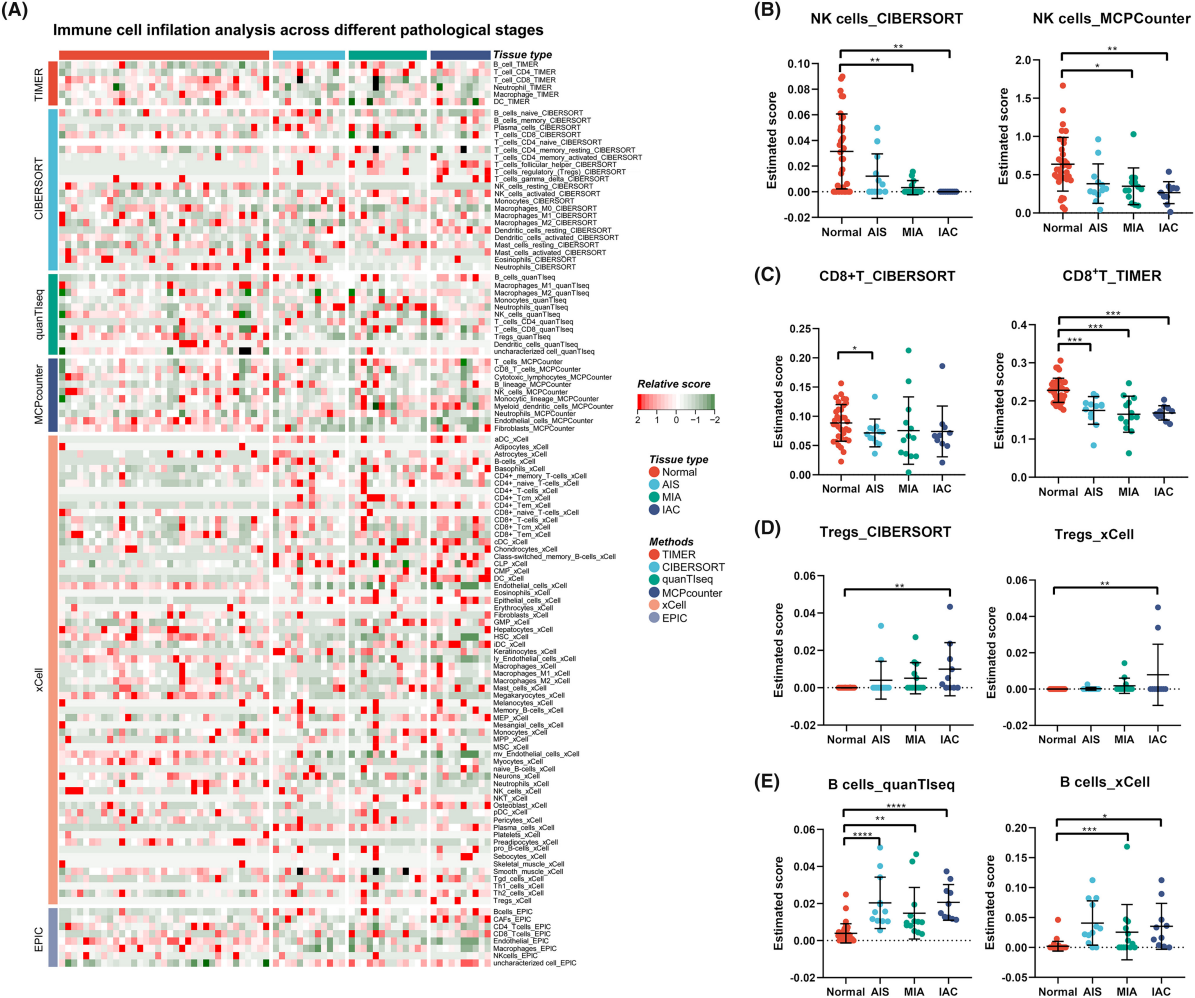
Immune cell signatures across different pathological stages of malignant pulmonary nodule. (A) The heatmap shows the immune infiltration scores calculated by six methods based on RNA‐Seq data of different pathological stages of malignant pulmonary nodules. (B) Scatter plot of NK cells immune scores for each pathological subtype. (C) Scatter plot of CD8+ T cells immune scores for each pathological subtype. (D) Scatter plot of Tregs immune scores for each pathological subtype. (E) Scatter plot of B cells immune scores for each pathological subtype.

To further study the relationship between the immune microenvironment and the invasive progression of LUAD, immune cell expression profiles clustered into normal, AIS, MIA, and IAC groups were analyzed using six calculation methods for the immune microenvironment score. Owing to the heterogeneity of the parsing algorithm, we summarized the most consistent tendencies of the six methods. As shown in Figure 9, the infiltration of natural killer (NK) cells was markedly reduced as the pulmonary nodules progressed in the CIBERSORT and MCPcounter results (Figure 9B). Similarly, CD8^+^ T cells were reduced in malignant nodular tissue, as determined by TIMER and CIBERSORT analyses (Figure 9C). In addition, regulatory T cells (Tregs) gradually increased as the pulmonary nodules progressed in CIBERSORT and xCell (Figure 9D). Interestingly, the B‐cell population increased in the tumor tissue according to the scores estimated by quanTIseq and xCel (Figure 9E).

## 4 DISCUSSION

GGNs signify the initial stages of lung cancer development,^20^, ^21^ and the molecular characteristics linked to GGNs may mirror the molecular processes associated with the early onset of lung carcinogenesis. High‐throughput sequencing technology has significantly advanced our comprehension of early LUAD biology, translating a wealth of molecular insights into novel therapeutic targets and biomarkers. This progress points toward enhancing the prognosis for patients. In recent years, numerous studies have individually explored the mutational and transcriptomic landscape of LUAD and identified enriched pathways specific to each. However, there is a lack of correlation analysis between these two data. For instance, Martinez‐Ruiz et al^22^ focused on describing the inheritance and transcription of lung cancer from early to advanced stages, Zhang et al^23^ provided a detailed genome picture of early LUAD, and Teixeira et al^24^ examined the genomic and epigenomic characteristics of pre‐invasive lung cancer. Although there are some reports on single‐cell sequencing of GGNs, these articles typically compare GGNs with solid nodules^25^ or advanced lung adenocarcinoma,^26^ or include patients with multiple GGNs.^27^ However, these data and comparative results do not seem to fully explain the molecular characteristics of the malignant transformation of solitary GGNs into early‐stage lung adenocarcinomas. In this study, we employed DNA and RNA‐Seq techniques to characterize solitary GGNs, which provided molecular evidence supporting the proposed model of early carcinogenesis in LUAD, from AIS to IAC. Furthermore, our analysis revealed evidence of progressive genomic evolution at SMGs and DEGs, accompanied by macroevolution during the transition from AIS to IAC.

The occurrence of somatic mutations in cancer genomes can be attributed to multiple mutational mechanisms, each of which gives rise to a specific mutational signature.^28^ The examination of mutational signatures is becoming a regular practice in the field of cancer genomics and carries important meanings for pathogenesis, categorization, evolution, and prognosis. The predominant base changes we observed within most GGNs, as recorded in the COSMIC signature database, were C > T or C > A mutations with high frequency. Our results show a significantly higher frequency of signature A in MIA and IAC compared to AIS. Additionally, signature A exhibits a high similarity with COSMIC signature 25 and 22, with Signature 22 being associated with the dynamic interplay of risk factors and cellular processes during tumorigenesis.^29^ The analysis of mutational signatures, to some extent, revealed a correlation between somatic mutations and evolution from AIS to IAC.

One of the central findings of this study was the identification of common mutations in LUAD presenting as GGNs, with EGFR mutations being the most prevalent, occurring in almost half of the patients studied. This aligns with previous research highlighting the significance of EGFR mutations in LUAD.^30^, ^31^ EGFR‐targeted therapies, such as tyrosine kinase inhibitors (TKIs), have shown promise in treating LUAD presenting as GGNs because of elevated EGFR mutation rates. Some researchers have begun exploring the use of EGFR‐TKIs for the treatment of multiple GGNs,^32^, ^33^, ^34^ with an observed efficacy rate of approximately 40%,^35^ which coincides with the prevalence of EGFR mutations in GGNs. In addition to EGFR, RBM10 mutations were found to be associated with progression from AIS to IAC. RBM10 encodes an RNA‐binding protein that has been implicated in cancer development and may serve as a potential target for further investigation.^36^, ^37^ We observed that RBM10 mutations are frequently accompanied by EGFR mutations, and studies suggest that RBM10 mutations can promote resistance to EGFR‐TKIs and have an impact on the survival of patients.^38^, ^39^ Furthermore, the EGFR/RBM10 co‐mutation group showed different mRNA expression patterns and slightly poorer survival than the group with only EGFR mutations. Our findings revealed a close relationship between EGFR/RBM10 co‐mutations and cholesterol metabolism, suggesting that cancer‐related signals promoting malignant progression can also regulate cholesterol metabolism. In the future, it may be possible to assess tumor malignant progression by detecting cholesterol metabolism through peripheral blood tests. In the CMap analysis, 10 compounds were identified as potential inhibitors of the pro‐oncogenic effects mediated by EGFR/RBM10 co‐mutations. Of these, W13 is a potent MsbA inhibitor that induces opposite effects on adenosine triphosphate (ATP) hydrolysis and has shown high efficacy in inhibiting tumor growth.^19^ Interestingly, recent research has provided evidence that W13 can block the PI3K‐Akt‐mTOR signaling pathway to inhibit colony formation, migration, and invasion in gastric cancer,^40^ indicating that W13 may have antitumor effects in the EGFR/RBM10 co‐mutation group. Therefore, understanding the role of RBM10 in LUAD progression may lead to the development of novel therapeutic strategies. Surprisingly, no KRAS mutations were detected in this study, although it has been reported that KRAS mutations occur in approximately 10%–25% of LUAD cases presenting as solid nodules.^41^ This finding suggests that GGNs may have mutation characteristics that differ from those of conventional lung cancer. Moreover, an increasing trend in the TMB was observed with pathological advancement, albeit without statistical significance. Therefore, characterization of larger cohorts is necessary to decipher common genomic evolutionary patterns from preneoplasia to invasive lung cancer.

We identified a panel of DEGs associated with the entire spectrum of invasive LUAD, encompassing the transition from AIS to IAC. These DEGs encompass a wide range of biological functions, and may serve as diagnostic markers, prognostic indicators, or therapeutic targets. Notably, GPR143, CCR9, ADAMTS16, and others were among the identified DEGs. Understanding the roles of these genes in LUAD biology is crucial to elucidate the molecular mechanisms underlying the disease progression. Numerous investigations have substantiated that CCR9 holds promise as a tumor biomarker for diagnostic and therapeutic purposes owing to its markedly increased expression in a diverse spectrum of malignant conditions. CCR9, upon activation by its selective ligand CCL25, can engage in multiple signaling pathways, particularly those implicated in tumor chemoresistance and metastasis.^42^, ^43^ In addition, mAb 92R, an anti‐CCR9 monoclonal antibody, has been discovered to synergistically enhanced the effects of chemotherapeutic agents, resulting in prolonged survival.^44^ Pathway analysis revealed alterations in the signaling pathways related to cell migration, invasion, and angiogenesis. The upregulation of the pathways associated with cell migration and invasion is consistent with the aggressive nature of invasive cancers. Conversely, the downregulation of angiogenesis‐related pathways may indicate an adaptive response by the tumor to reduce its reliance on angiogenesis, potentially contributing to its invasive behavior. Overall, these findings suggest potential therapeutic avenues, such as targeting invasion‐related pathways or developing antiangiogenic strategies for LUAD treatment.

We demonstrated a significant correlation between mRNA expression levels and CNVs for specific genes. This observation highlights the intricate relationship between genetic alterations and gene expression in patients with LUAD. Genes such as FAM83A, MAL2, and DEPTOR, which exhibit strong correlations, may play functional roles in disease progression. These genes are attractive candidates for further investigation and potential therapeutic interventions. Family with sequence similarity 83A (FAM83A) has been described as a novel oncogene in numerous human cancer specimens and is associated with a poor prognosis.^45^, ^46^ Hu et al^47^ demonstrated that FAM83A promotes tumorigenesis in NSCLC, at least partly via the ERK and PI3K/Akt/mTOR pathways. MAL2 has been implicated in the pathogenesis of several malignancies. Jeong et al^48^ demonstrated that MAL2 is crucial for lipid raft formation, HER2 signaling, and HER2 membrane stability in breast cancer cells. In future studies, we may construct a diagnostic model based on the genes detected which continuously increase with the pathological stage, providing a basis for early lung cancer diagnosis and treatment.

Currently, our understanding of the formation of immune microenvironments as ground‐glass opacities in LUAD is limited. Immune microenvironments can vary not only between different tumors but also within tumors and across various subtypes during tumor evolution. Furthermore, tumor tissues have a distinct immune microenvironment compared to normal tissue. The presence of significantly decreased NK and CD8^+^ T cells, as well as an increase in Treg/B cells, in tumor tissues suggests that the immune response mediated by CD8^+^ T/NK cells is suppressed, allowing for tumor cell dissemination and survival by inhibiting immune‐mediated elimination. Simultaneously, a recent study has found that depletion of MAL2 in breast tumor cells profoundly enhanced the cytotoxicity of tumor‐infiltrating CD8^+^ T cells and suppressed breast tumor growth,^49^ suggesting that MAL2 is a potential therapeutic target for immunotherapy. Moreover, the number of secondary lymph nodules in tumor tissue has been found to be positively correlated with corresponding immunotherapy.^50^, ^51^ However, immunotherapy appears to be ineffective for GGNs,^52^ potentially indicating that mature secondary lymph node structures might not have formed despite increased B‐cell infiltration.

This study examined a cohort of 35 individuals diagnosed with LUAD presenting as ground‐glass opacities, focusing on sex distribution, smoking habits, and age of onset in relation to pathological progression. We revealed a notable gender imbalance, with only eight males in the cohort. Females have been identified as a high‐risk population for this type of cancer, although the specific underlying reasons remain undetermined. This observed disparity may be linked to the hormonal levels. Additionally, a history of smoking was prevalent in merely four individuals, suggesting that smoking might not be directly associated with the incidence of ground‐glass opacity lung cancer. Moreover, no significant relationship was found between the pathological stage and onset age.

Despite these findings, this study had several limitations. First, we examined a small number of patients for each histological subtype. We selected only 35 patients from our lung cancer cohort who met our sample criteria, and the limited sample size diminished the statistical power in our stratified analysis. Therefore, additional samples are necessary to validate the findings of this study further. Second, the assessment of dynamic tumor evolution in this study relied on bulk cell RNA‐Seq analysis. While more recent technologies like single‐cell transcriptome analysis are available, the retrospective nature of our study prevented us from obtaining tissues that were specifically preserved and suitable for single‐cell analysis. As a result, it was challenging to verify the correlation between the molecular properties of the tumor and clinical treatment. Finally, the predominance of female and non‐smoker patients in this study could introduce potential confounding factors when considering the prevalence of transitions versus transversions. These challenges will be carefully examined in future investigations, and the knowledge acquired from the present study will guide us in implementing appropriate patient stratification strategies.

In summary, our data revealed a new invasion‐associated evolutionary pattern at the somatic SNV, CNVs, and mRNA expression levels; revealed the immune microenvironment in GGNs; and explored the molecular mechanisms between CNVs and DEGs. Our results provide a new experimental basis to understand early stage LUAD.

## AUTHOR CONTRIBUTIONS

**Dong Zhou:** Methodology (lead); writing – original draft (lead). **Yan‐qi Li:** Data curation (lead); resources (lead). **Quan‐xing Liu:** Conceptualization (equal); data curation (equal). **Xu‐feng Deng:** Data curation (equal). **Liang Chen:** Data curation (equal); formal analysis (equal). **Man‐yuan Li:** Investigation (equal); resources (equal). **Jiao Zhang:** Data curation (equal); formal analysis (equal). **Xiao Lu:** Conceptualization (equal); formal analysis (equal). **Hong Zheng:** Conceptualization (lead); writing – review and editing (lead). **Ji‐gang Dai:** Project administration (lead); writing – review and editing (equal).

## FUNDING INFORMATION

This study was supported by grants from the National Natural Science Foundation of China for Dai (No. 81972190), the Key Projects on Technological Innovation and Application Development of Chongqing (2022‐195), The Science and Technology Commission and the Chongqing Health Commission Joint Medical Research Program of Chongqing (2024MSXM090), and the Excellent Talent Program of Chongqing (cstc2022ycjh‐bgzxm0109).

## CONFLICT OF INTEREST STATEMENT

None of the authors have a financial or conflict of interest in the outcome of this research.

## ETHICS STATEMENT

The study protocol was approved by the Ethics Committee of the Xinqiao Hospital, Army Medical University, Chongqing, China (#2020‐144‐01).

## Data Availability

All the data in the paper are free to obtain. Raw data that support the findings of this study are available from the corresponding author upon reasonable request.
